# Disseminated Pelvic Actinomycosis Caused by *Actinomyces Naeslundii*

**DOI:** 10.3390/antibiotics9110748

**Published:** 2020-10-29

**Authors:** Olga Džupová, Jana Kulichová, Jiří Beneš

**Affiliations:** 1 Department of Infectious Diseases, Third Faculty of Medicine, Charles University, 100 00 Prague, Czech Republic; benes.infekce@seznam.cz; 2 Department of Infectious Diseases, Hospital Na Bulovce, 100 81 Prague, Czech Republic; jana.kulichova@volny.cz

**Keywords:** pelvic actinomycosis, disseminated actinomycosis, intrauterine device, *Actinomyces naeslundii*

## Abstract

Actinomycosis is a chronic bacterial infection characterized by continuous local spread, irrespective of anatomical barriers, and granulomatous suppurative inflammation. Due to its expansive local growth, it can simulate a malignant tumour. Subsequent hematogenous dissemination to distant organs can mimic metastases and further increase suspicion for malignancy. A case of severe disseminated pelvic actinomycosis associated with intrauterine device is described here. The patient presented with a pelvic mass mimicking a tumour, bilateral ureteral obstruction, ascites, multinodular involvement of the liver, lungs and spleen, inferior vena cava thrombosis and extreme cachexia. Actinomycosis was diagnosed by liver biopsy and confirmed by culture of *Actinomyces naeslundii* from extracted intrauterine contraceptive device (IUD). Prolonged treatment with aminopenicillin and surgery resulted in recovery with moderate sequelae.

## 1. Introduction

Actinomycosis is a disease with characteristic features, including a slow growth of a solid mass arising from local massive fibroproduction, spread across tissues regardless of natural barriers resembling a malignant tumour, formation of abscesses and fistulas, and growth of bacteria in the colonies surrounded by granulomatous inflammation, macroscopically described as sulphur granules [1].

Actinomycosis can usually be successfully treated with long-term antibiotic therapy. The main challenge is to make an early correct diagnosis because the disease begins discreetly and the patient’s troubles are nonspecific, especially when internal organs are involved.

Traditionally, three forms of the disease have been described, depending on the location: cervicofacial, thoracic and abdominal—the last one originates in the bowel. After intrauterine devices (IUD) had been introduced to prevent conception, the pelvic actinomycosis began to appear frequently. However, many authors do not recognize the pelvic form as a separate unit and report on abdominopelvic actinomycosis [2,3,4,5,6]. This approach is based on the idea that although intestinal actinomycosis and uterine actinomycosis have different pathogeneses, the clinical course is similar and differentiation in the advanced stage can be difficult.

Another terminological problem concerns the aetiology. Until 1990, only five species of pathogenic actinomycetes were known: *Actinomyces israelii*, *A. naeslundii*, *A. viscosus*, *A. odontolyticus* and *A. meyeri* [7]. As a result of the introduction of modern diagnostic methods based on genotyping, another twenty potentially pathogenic species were described by 2010, the most important of which are *A. gerencseriae*, *A. graevenitzii*, *A. turicensis*, and *A. neuii* [1,7]. Therefore, the species identified in studies and case reports older than 10 years may be questioned.

*Actinomyces naeslundii* was described in the 1950s [8,9]. It was considered a saprophyte of the human mouth and occasional agent of periodontal disease and dental root caries [10,11]. This role is now assigned to the new species *Actinomyces oris* that was separated from *A. naeslundii* in 2009 [12]. The ability of *A. naeslundii* to cause actinomycosis in various organs and tissues has been known since 1969 [13]. Bonnez et al. first reported on *A. naeslundii* causing pelvic actinomycosis [14].

We describe a female patient with disseminated actinomycosis secondary to IUD. The case indicates the ability of this infection to progress slowly with only general non-specific symptoms to an advanced stage imitating a malignant metastatic gynaecological tumour.

## 2. Case Presentation

Ethical statement: The patient´s written and signed consent to publish the case report was obtained and provided to the editor.

A 35-year-old female with a history of no chronic comorbidity, divorced and living single with her two school-age children, was referred to the hospital with weight loss and fatigue lasting for almost 6 months. She did not seek help earlier because she was afraid of job loss and later of being diagnosed with a malignant disease. She had an intrauterine contraceptive device (IUD) which was inserted 8 years ago. On admission, she presented with severe cachexia, prominent ascites, hepatomegaly, and oral candidiasis. She was conscious and cooperative, afebrile, eupnoeic, with heart rate 83 b/min and blood pressure 104/57 mm Hg.

Laboratory results showed hypochromic microcytic anaemia, leukocyte count 11.2 × 10^9^/L with 86% neutrophils, D-dimers >2000 ng/mL, urea 12.5 mmol/L, creatinine 178 μmol/L, clearance 0.873 mL/s, albumin 16 g/L, prealbumin 0.08 g/L, C-reactive protein 115 mg/L, alkaline phosphatase 7.24 µkat/L, gamma-glutamyltransferase 4.74 µkat/L, cholinesterase 24 µkat/L (reference range (RR) 71–187), ferritin 1653 pmol/L (RR 4.6–204), and IgG 37.8 g/L (RR 7–16). Other biochemical tests results, as well as lymphocyte subpopulations, were in the normal range. Screening of autoantibodies was negative. 

**Figure 1:**
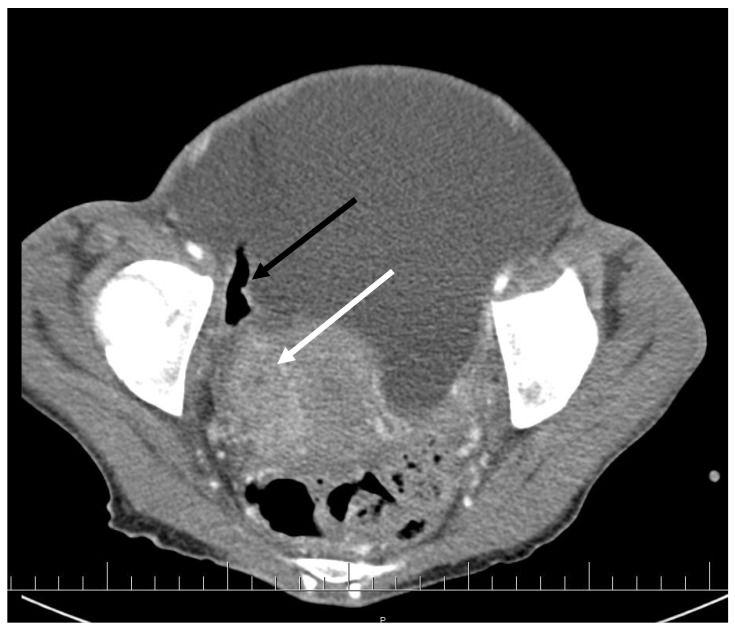
Pelvic CT scan shows ascites, enlarged right ovary (white arrow) and a gas-containing non-homogenous mass adhering to the uterus (black arrow).

**Figure 2:**
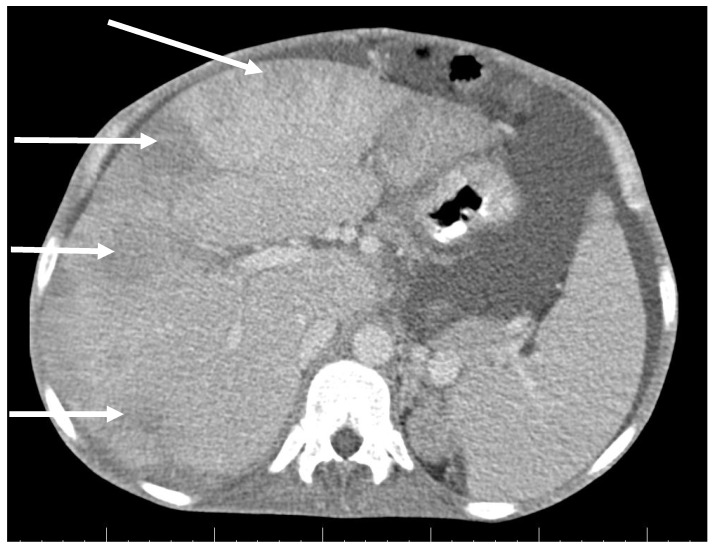
Abdominal CT scan shows multiple lesions in the liver (arrows).

CT scan showed ascites, an enlarged right ovary with a parauterine non-homogenous mass 8 × 5 cm in size containing gas, bilateral hydronephrosis, ischaemic foci in spleen, multiple bilateral tiny nodules in the lungs with mainly subpleural distribution resembling miliary tuberculosis or multiple emboli, multiple liver lesions described as signs of Budd–Chiari’s syndrome due to the inferior vena cava thrombosis, and right hepatic vein thrombosis (Figure 1 and Figure 2).

The interferon-gamma release assay (QuantiFERON-TB Gold) was negative as well as HIV 1 and 2 antibodies. Angiotensin-converting enzyme activity was in the normal range. One set of blood culture and ascitic fluid was sterile. Gastroduodenoscopy showed erosive oesophagitis and gastritis with reflux disease Grade I-II.

The diagnosis of metastatic ovarian cancer was assumed. Histology of a CT-guided fine-needle liver biopsy sample revealed fibroproliferative inflammation and chronic abscess with actinomycotic granules. 

Treatment with intravenous ampicillin 2 g q.i.d., metronidazole 0.5 g t.i.d. and oral fluconazole 100 mg b.i.d. was started, as well as supportive parenteral nutrition and treatment of hepatorenal failure. Bilateral nephrostomy was inserted because of hydronephrosis due to ureteral compression by pelvic mass. The IUD was removed; on microscopy, branching Gram-positive rods were seen. *Actinomyces naeslundii*, sensitive to penicillin, ampicillin, clindamycin, chloramphenicol, cefoxitin, erythromycin, and metronidazole, *Bacteroides* sp. and *Escherichia coli* were identified by culture. The oral cavity swab culture rendered *Candida albicans*, but no *Actinomyces*.

Transthoracic echocardiography (TTE) showed no signs of endocarditis but thrombosis of the inferior vena cava reaching the right atrium and tricuspid valve. It could not be removed by catheterization; therefore, heparin anticoagulation was given. 

**Figure 3:**
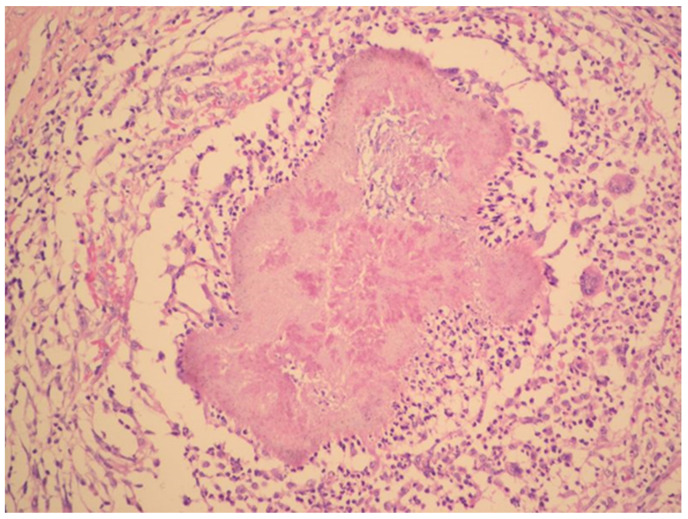
Histopathology shows granuloma with purulent exudate and actinomycotic granules. Haematoxylin–eosin stain. (With kind permission of Dr. Kamila Benková, Department of Pathology, Hospital Na Bulovce.).

The radical surgery was postponed for two months until the patient´s nutritional status improved. Hysterectomy, bilateral salpingo-oophorectomy, appendectomy and adhesiolysis was performed. On the cut, the mass on the right ovary, with dimensions of 50 × 50 × 30 mm, showed yellowish tissue with necrosis. Histopathology showed that the mass consisted of confluent granulomas with purulent exudate containing fibrillar structures corresponding to actinomycotic granules despite two months of targeted antibiotic treatment (Figure 3).

Postoperative healing was uneventful. Follow-up CT two weeks after surgery showed no pelvic mass, decreased ascites, persisting pulmonary nodules and signs of Budd–Chiari´s syndrome. A right-sided pleural effusion was punctured with negative culture and 16S panbacterial PCR. Left-sided nephrostomy was removed but the right-sided remained necessary as the ureteral obstruction persisted and intraluminal stenting was unsuccessful. The caval thrombus slowly decreased its size. Intravenous antibiotics were given for 94 days in total. Renal and liver functions slowly improved, as well as nutritional status. The patient was discharged home on day 96 with oral amoxicillin 1 g t.i.d. and warfarin.

Nephrostomy catheter was removed 3 months later. At the follow-up visit 6 months after the discharge, she had gained 10 kg, laboratory results showed chronic kidney disease stage 3 with moderately decreased function and TTE finding was normal with no signs of thrombus. Antibiotic therapy was finished. At the 12-month follow-up visit, CT showed liver remodelling with incipient cirrhosis, persisting miliary nodules in the lungs decreasing in quantity, and mild right-sided ureteral dilatation. Secondary osteoporosis was treated with oestrogen substitution. The patient was able to take care of herself and her children and received a disability pension. She still complained of weakness and tiredness. Three years later, she remained free of relapse.

## 3. Discussion

Pelvic actinomycosis is associated with the use of an IUD in the majority of reported cases [5,15,16,17,18,19]. The common scenario shows a female admitted with a diagnosis of a malignant pelvic tumour based on the finding of ultrasonography or CT. The patient undergoes diagnostic and/or palliative surgery and actinomycosis is diagnosed by histological examination of the excised tissue. The infection is not considered initially because patients have no clinical or laboratory signs of severe bacterial infection, such as fever, leucocytosis with left-shift, elevated C-reactive protein and procalcitonin levels.

Our case report describes several peculiarities. The infection manifested as prolonged weakness, general exhaustion and progressing cachexia, while the local signs from the affected pelvic and abdominal organs became apparent only at a very advanced stage. In addition, hematogenous dissemination to distant parenchymal organs occurred, mimicking metastases of a malignant tumour. The initial diagnostic consideration was malignancy, tuberculosis or HIV infection. The diagnosis of actinomycosis in this case was primarily based not on histological examination of excised tumour mass but on the histology of a liver biopsy sample wherein the biopsy was indicated to determine the type of tumour. The etiological agent was then isolated from an extracted IUD. 

Disseminated actinomycosis is defined as a disease affecting at least two distant organs—in other words, hematogenous spread is expected [20]. In our patient, the infection probably contiguously spread from the uterus to the adnexa, ureters and adjacent bowel loop, parietal peritoneum and inferior vena cava. The lungs, spleen and liver were probably affected via the bloodstream. Disseminated actinomycosis was reported to be mostly due to *A. meyeri* [21,22]. To the best of our knowledge, disseminated actinomycosis due to *A. naeslundii* has not been described yet.

Antibiotic treatment of actinomycosis must be prolonged. Intravenous penicillin G is generally recommended as the drug of choice. We prefer aminopenicillins because of better pharmacokinetics, i.e., a longer half-life allowing dosing 3–4 times daily. Additionally, it can be switched early to oral amoxicillin, which is convenient for its good intestinal absorption. Our patient was treated for 9 months in total. Her general practitioner respected the recommendation to treat a severe form of the disease for 6–12 months [1] and was afraid to finish sooner. In our opinion, the length of antibiotic treatment should be guided by remission of actinomycotic infiltrates demonstrated by imaging. In this case, a six-month course of treatment would have been sufficient.

In retrospect, the extent of surgery in our patient may be considered too radical. The uterus and right adnexa were too severely affected. The fact that the patient already had two children, her health condition was poor, and her social situation was unsatisfactory also played a role in the planning of the surgery. However, the left ovary may have been preserved and subsequent oestrogen substitution would not be necessary.

Renal involvement was caused by bilateral ureteral stricture which we repeatedly encountered in patients with pelvic actinomycosis. If a single ureter is affected and the disease is oligosymptomatic, it may be diagnosed late and result in severe kidney damage.

Although the patient lived under long-term mental stress before the onset of the disease, she had no signs of impaired immunity, and laboratory tests did not show any immune deficiency. Impaired immunity is unlikely to present an increased risk of actinomycosis. It is an anaerobic infection, and these occur primarily in tissue that is injured or ischemic. The standard immune response has little, if any, effect on the onset and spread of these infections.

In conclusion, pelvic actinomycosis should be considered in any woman with a history of IUD use presenting with a pelvic or abdominal tumour-like mass, especially if the IUD is left in situ for more than the recommended period of 5 years [17,19]. Immediate extraction of the IUD and its culture, as well as antibiotic treatment with intravenous benzylpenicillin or ampicillin, is recommended. The timing and extent of surgery should be guided by gynaecologist. In the case of highly suspected or confirmed actinomycosis, the radicality of surgery, if indicated at all, should be limited to what is strictly necessary, as the disease can be successfully treated with antibiotics, even in the advanced stage.
